# Autism spectrum disorder and the role of nuclear hormone receptors: insights and therapeutic implications

**DOI:** 10.3389/fnins.2025.1655348

**Published:** 2025-10-30

**Authors:** Shivakanth Chintalapally, Kalpana Rajanala, Arun Upadhyay

**Affiliations:** ^1^Ocugen India, Hyderabad, Telangana, India; ^2^Ocugen, Malvern, PA, United States

**Keywords:** autism spectrum disorder, NHRs, RORA, adeno-associated viral vectors, gene therapy

## Abstract

Autism spectrum disorder is a neurological and developmental condition known to impact a person's learning, communication, and interpersonal interactions. Recent research has highlighted the role of nuclear hormone receptors (NHRs) in neurodevelopment and synaptic function, suggesting their potential involvement in ASD pathophysiology. NHRs regulate gene expression that are critical for neural differentiation, plasticity, and metabolic processes. Dysregulation of these receptors can lead to altered neural circuit formation and neurotransmitter imbalances, which are commonly observed in ASD. Understanding the interplay between NHRs and ASD could open new avenues for therapeutic interventions, providing hope for more personalized approaches to managing the disorder. One key receptor is retinoic acid-related orphan receptor-alpha (RORA), which was shown to be reduced in individuals with ASD. Among its numerous functions during development, RORA was shown to regulate the transcription of genes involved in neuronal differentiation, synaptic function, and neuroprotection. Studies have identified that RORA expression is reduced in individuals with ASD, particularly in the prefrontal cortex and cerebellum, affecting transcription of multiple ASD-associated genes. In the present review, we discuss the underlying mechanisms leading to ASD pathophysiology, various treatment modalities, the prospects of the RORA gene therapy approach, and future perspectives.

## Introduction

Autism is referred to as a “neurodevelopmental disorder” since symptoms often manifest in the first 2 years of life, even though a diagnosis can be made at any age. Differences in social and communicative behaviors, intellectual difficulties, and other physical and mental health concerns are just a few of the many symptoms that people with autism may experience, hence, it is considered a spectrum disorder. Autism has a heterogeneous diagnosis that may undergo developmental changes over time and an array of epidemiologic, genetic, epigenetic, and environment-related factors has been associated with the condition. Among the additional characteristics are atypical patterns of activities and behaviors, as demonstrated by challenges in switching between activities, a strong focus on minute details, and atypical responses to sensory input. Intellectual disability [currently estimated at around 30% of cases (Christensen et al., 2012); historically estimated at around 70%] and attention deficits (occurring in approximately 30%−40% of cases, though estimates outside of this range are common) are common impairments that correspond to autism spectrum disorder (ASD). Other common impairments include sensory sensitivity, gastrointestinal issues, immunological deficiencies, anxiety and depression, sleep disturbances, and a variety of comorbid medical conditions (Croen et al., 2015; Matson and Cervantes, 2014). Although some people with autism are capable of leading independent lives, others require lifelong care and support. Opportunities in education and employment are often impacted by autism. Early intervention and tailored support can significantly improve outcomes for individuals with the condition. It is therefore crucial to recognize and accommodate the unique strengths and challenges of each person, promoting inclusion and understanding in all aspects of life.

Based on research on autism prevalence around the world, considering how socioeconomic, racial, and geographic factors affect prevalence estimates, worldwide, about 1 in 100 children receive a diagnosis of autism spectrum condition. According to estimates in the US, one in 36, 8-year-old children (about 4% of boys and 1% of girls) will have ASD in 2020 (Maenner et al., 2023). Over the past three decades, the prevalence of ASD has gradually but significantly risen across Europe, North America, and Oceania (Anorson et al., 2021). For children of about 4 years, the prevalence increased by 26% from 2018 to 2020 (Shaw et al., 2023). The prevalence estimate in 2020 for children in the age group of 8 years was 27.6% (Maenner et al., 2023). Sex related bias has been observed in the prevalence of ASD where there exist roughly four males for every girl with ASD. This “male to female” sex ratio seemed to decline with increasing severity of ASD (Werling, 2013). Females often camouflage their autistic traits by mimicking social behaviors, suppressing stimming, or rehearsing conversations, which can lead to underdiagnosis (Wood-Downie et al., 2021), and thereby significantly impacts the male-to-female incidence ratio (Cancino-Barros and Villacura-Herrera, 2025). Historically, there has been a substantial sex gap evident in all populations under study (Dworzynski et al., 2012), the variations in how symptoms appear in females and the potential diagnostic biases (Dworzynski et al., 2012) need further research.

In this review, we discuss the key characteristic features of ASD, genetic and molecular pathways, the role of nuclear hormone receptors, aspects related to gen therapy, and other therapeutic interventions.

## Characteristic features and pathophysiology

A higher incidence of ASD in the offspring has been linked to maternal use of pharmaceutical medications, particularly in the first trimester of pregnancy, such as thalidomide, valproic acid, and antidepressants (primarily selective serotonin reuptake inhibitors; Croen et al., 2011; Christensen et al., 2013). Distinguishing the effects of medicine from those of the mother's underlying disease, which may potentially affect autism risk, can be challenging (Lyall et al., 2013). Particularly for those who are genetically predisposed, and have exposure to a variety of toxicants, such as pesticides, polychlorinated biphenyls (PCBs), and polybrominated diphenyl ethers (PBDEs), can negatively impact developmental processes. In addition to their neurotoxic and endocrine-disrupting properties, PCBs and PBDEs also bioaccumulate in the food chain and remain in the environment (Newschaffer et al., 2007). Furthermore, several neurotoxic substances may disrupt neurotransmitter systems linked to ASD (Quaak et al., 2013). Pregnancy-related ASD risk has been linked to maternal residential proximity to agricultural pesticide applications; however, this situation might indicate abnormally high exposure levels (Ornoy et al., 2015). Additionally, these substances may be immunotoxic, which could result in the changed cytokine production commonly seen in ASD (Goines and Ashwood, 2013). A recent worldwide study confirmed that older parents are known risk factors for autism, but it also indicated that parents who are not of the same age increase the likelihood of developing ASD (Sandin et al., 2016). It is believed that methylation abnormalities in gametes, which can be brought on by elevated oxidative stress that damages and fragments DNA, are a result of older parents (Menezo et al., 2015). Obesity, diabetes, and folic acid insufficiency are maternal metabolic and nutritional risk factors. Zinc deficiency has been observed in autistic children and may play a role in pathogenesis (Lyall et al., 2013; Grabrucker, 2013; Ornoy et al., 2015).

Intellectual disability, motor impairment, attention issues, sensory differences, sleep disorders, seizures, and externalizing behaviors like violence and affective disorders are among the co-morbid conditions that are linked to ASD (Maski et al., 2011; Devnani and Hegde, 2015). Rett syndrome, Asperger syndrome, autism, and pervasive developmental disorder-not otherwise specified are among the many complex and varied conditions that are included in the category of autism spectrum disorders (Barrett et al., 1996). Although monogenic syndromes do not always have traits of autism spectrum disorder (ASD), many do have a high prevalence of ASD diagnoses or traits. The expression of ASD traits depends on the specific syndrome, the gene involved, and personal traits such as sex and mutation type (Lord et al., 2000). Studies in toddlers infer that a deficiency in social interaction, communication, and behavior may indicate early signs of autism as early as 14 months of age, and clinical signs appear at 3 years (Landa et al., 2007). Numerous studies show that some disorders with predisposing genetic syndromes are closely associated with ASD (Jedele, 2007; Autism Research Institute, 2023, 2022; White et al., 2020; Ornoy et al., 2016; Genovese and Butler, 2023). Both genetic and environmental factors contribute to the pathophysiology of ASD, even if the causes of these anomalies are still being studied (Herbert et al., 2006; Minshew and Williams, 2007; Persico and Bourgeron, 2006).

Although the etiologies of most patients are still unknown, ASD exhibits clinical variability and can co-occur in up to 10% of cases with well-characterized neurological and genetic abnormalities, including co-occurring syndromes (Rapin, 2002). Some academics support using the term “autism” rather than a single disorder because of the clinical unpredictability and heterogeneity linked to autism (Geschwind and Levitt, 2007). Though monogenic pathologies such as Angelman syndrome, Rett syndrome, tuberous sclerosis, or Phelan–McDermid syndrome, etc. have different pathologies, researchers argue that mutation discovery of this sort offers an important opportunity to identify neurodevelopmental mechanisms of the disease. They hope that these mechanisms will show some degree of convergence that may be amenable to treatment intervention (Gamsiz et al., 2015).

**Figure 1:**
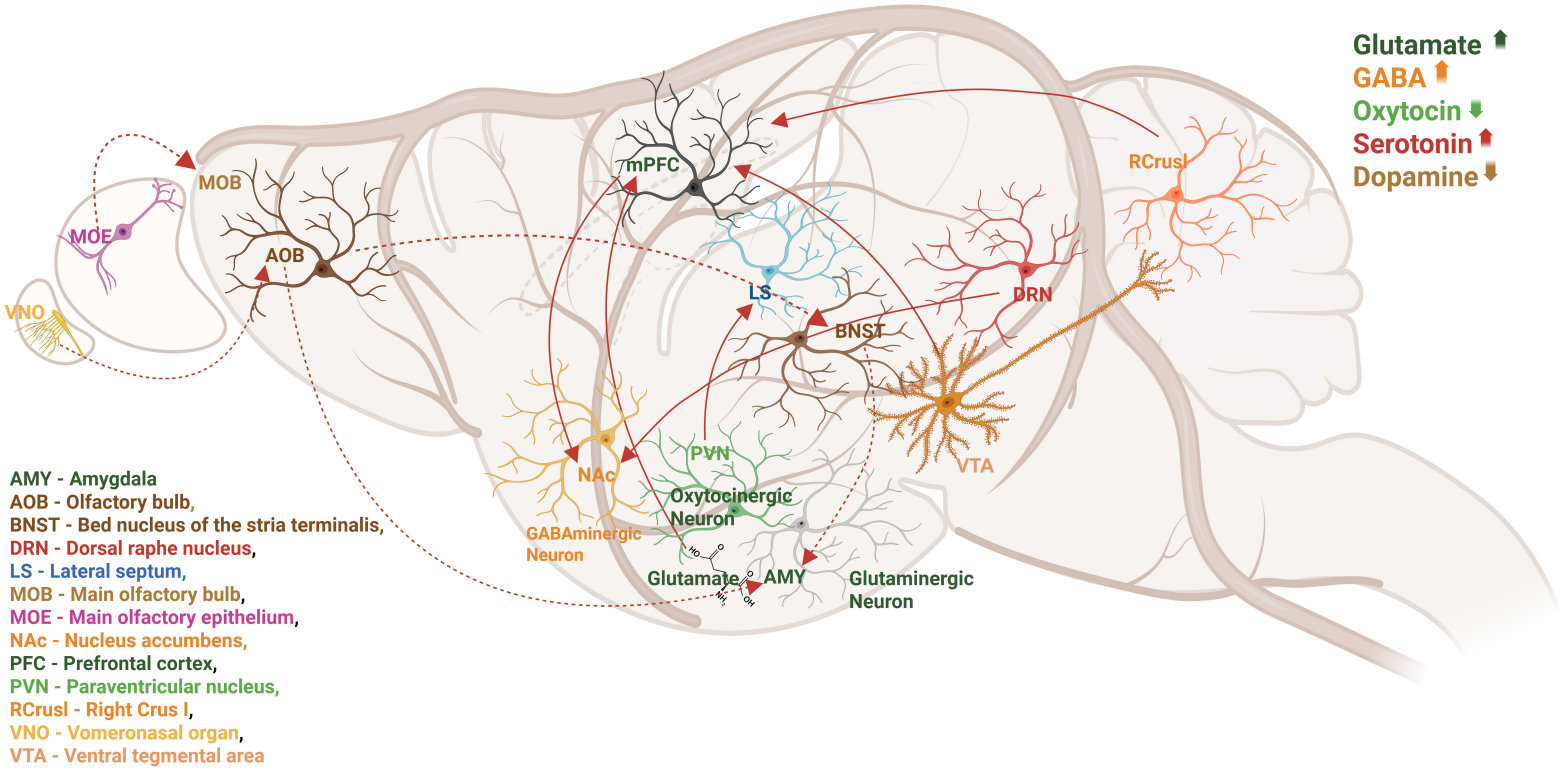
Local and distal circuits involved in social behaviors are depicted in a sagittal image of the rodent brain. Numerous neurotransmitters and neuromodulators collaborate to coordinate the activity of brain regions and neural circuits. Studies indicate that autism is linked to disruptions in neurotransmitter systems, including glutamate, GABA, serotonin (5-hydroxytryptamine, 5-HT), melatonin, dopamine (DA), OT, and arginine vasopressin (AVP). Therefore, dynamic variations in neurotransmitter concentration, release, and receptor density may directly impact behavioral performance and neuronal circuit function.

Autism is linked to disruptions in neurotransmitter systems, including those involving glutamate, GABA, serotonin (5-hydroxytryptamine, 5-HT; Pagan et al., 2014), melatonin (Melke et al., 2008), dopamine (DA; Farook et al., 2012) and arginine vasopressin (AVP; Figure 1). New behavioral neuroscience, chemical genetics, optogenetics, and electrophysiology methods have aided in developing connections between different social behaviors and cerebral circuit activity as shown in Figure 1.

Although they only make up a small percentage of cases, several genetic disorders are known to have strong correlations with autism. Monozygotic twins had approximately 91% autism heritability rate, which drops to 53% in dizygotic twins (Tick et al., 2016). Numerous associations between non-genetic factors and ASD have been found, which paved the way for additional research to examine mechanisms for the development of the disorder (Grabrucker, 2013).

### Genetic factors associated with ASD

Autism is the most heritable psychiatric illness (Hawi et al., 2018). Apart from genomic screening and candidate gene studies, the identification of chromosomal abnormalities and Mendelian syndromes in individuals with autism has helped refine our understanding of the complex genetics underlying these conditions. In a case series study, when only cases without minor congenital anomalies were included, the male-to-female sex ratio was nearly 10:1, and there was a much higher rate of autism cases in the families (Miles and Hillman, 2000). Though modern studies exclude cases with known etiologies, but do not exclude mild, dysmorphic features of unknown origin. For autism that the examiners judge to be idiopathic, the most parsimonious genetic model is one in which several genes interact with one another to produce the autism phenotype. A cytogenetic analysis of 67 people who had previously been diagnosed with mental retardation and autistic characteristics showed that 3 (4.5%) had autosome abnormalities and one person (1.5%) had the fragile X chromosome [fra(X); Cantú et al., 1990]. Autism, along with atypical bipolar disorder associated with 15ql2 deletion, was first reported by Kerbeshian et al. (1990). Partial trisomy of chromosome 15 may be linked to a distinct condition that includes mild-to-moderate physical stigma, autistic behavior, and moderate-to-severe mental impairment. Kyphoscoliosis, epilepsy, and muscular hypotonia seem to be related characteristics (Gillberg et al., 1991). The region 15pter-ql3, which has a molecular border at locus D15S24, has one or more genes that are predisposed to behavioral issues, including infantile autism or autistic disorder (Martinsson et al., 1996). ASD is linked to the chromosomal abnormality of an extra marker chromosome with a duplication of 15q11-13 and the clinical symptoms of epilepsy and ataxia (Bundey et al., 1994). The GABRA5 and GABRB3 genes on the maternally derived chromosome are duplicated, and an interstitial duplication of 15q (Bundey et al., 1994). Siblings with ASDs had mutations in the neuroligins NLGN3 and NLGN4, the two X-linked genes, which impact synapse-localized cell adhesion molecules, indicating synaptogenesis breakdown may be a risk factor for autism (Jamain et al., 2003). In addition to duplication, which also seems to be a high-penetrance risk factor, five cases of a *de novo* deletion of 593 kb on chromosomes 16p11.2 were found among Autism Genetic Resource Exchange (AGRE) families (Weiss et al., 2008). A comparative genomic hybridization, a reciprocal microduplication, and a unique, recurring microdeletion carry a significant risk of autism and are be responsible for around 1% of cases (Weiss et al., 2008). Durand and colleagues provide insight into one gene dosage-sensitive synaptic pathway implicated in autism spectrum disorders, although only a small percentage of people can develop language and/or social communication impairments due to a mutation of a single copy of SHANK3 on chromosome 22q13 (Durand et al., 2007). Following data analysis using an affected sib-pair (ASP) approach, the whole-genome microsatellite marker analysis of 110 multiplex families using 335 microsatellite markers produced multipoint maximum LOD scores (MLS) that surpass the recognized threshold for suggestive linkage on chromosomes 5, X, and 19 (Liu et al., 2001). The significance of peak LOD scores based on statistical evidence at adjacent marker loci was also evaluated using scan statistics to increase the power to detect linkage. The results showed impressive evidence for linkage to autism and autism-spectrum disorders, with significant genome-wide *P* values < 0.05 for markers on chromosomes 5 and 8 and suggestive linkage evidence for a marker on chromosome 19 (Liu et al., 2001). In 427 unrelated ASD cases, karyotyping and single-nucleotide polymorphism microarrays were performed to evaluate the genome for structural abnormalities. In 44% of ASD families, 277 imbalanced copy number variants (CNV), (i.e., the number of copies of a particular gene varies from one individual to the next) were identified using microarrays that were absent in 500 controls and additional balanced alterations were discovered using karyotyping where 27 cases with *de novo* modifications, and in three (11%) of these individuals, two or more new variations were recorded, though the majority of variants were inherited, additional genes (DPP6, DPP10 PCDH9, RPS6KA2, and RET); other hereditary CNVs (IDS, IL1RAPL1, and TSPAN7), MR loci (15q24, 16p11.2) implicate the SHANK3, NLGN4, and NRXN1-PSD genes (Marshall et al., 2008). Genetic abnormalities in ASD include *de novo* mutations (Sanders et al., 2012; O'Roak et al., 2012), Multiple genes are implicated in synaptic formation, transcriptional regulation, and chromatin-remodeling pathways (De Rubeis et al., 2014). Mutations in FMR1, SHANKs, NRXNs, NLGNs, SYNGAP1, CHD2, SCN2A, POGZ, ADNP, and DYRK1A share pathways with altered NMDAR (glutamate receptors; Zhang et al., 2024).

#### Molecular pathways involved in ASD

Dysregulated signaling and metabolic pathways that are indicative of a systemic illness that may be treated once identified are at least partially responsible for the neurological manifestations of ASD. The distinct biological phenotypes of ASD are corroborated by pathway analyses, which also distinguish several types of idiopathic autism symptoms. Studies have identified several pathways related to ASD, some of which are listed below.

**Figure 2:**
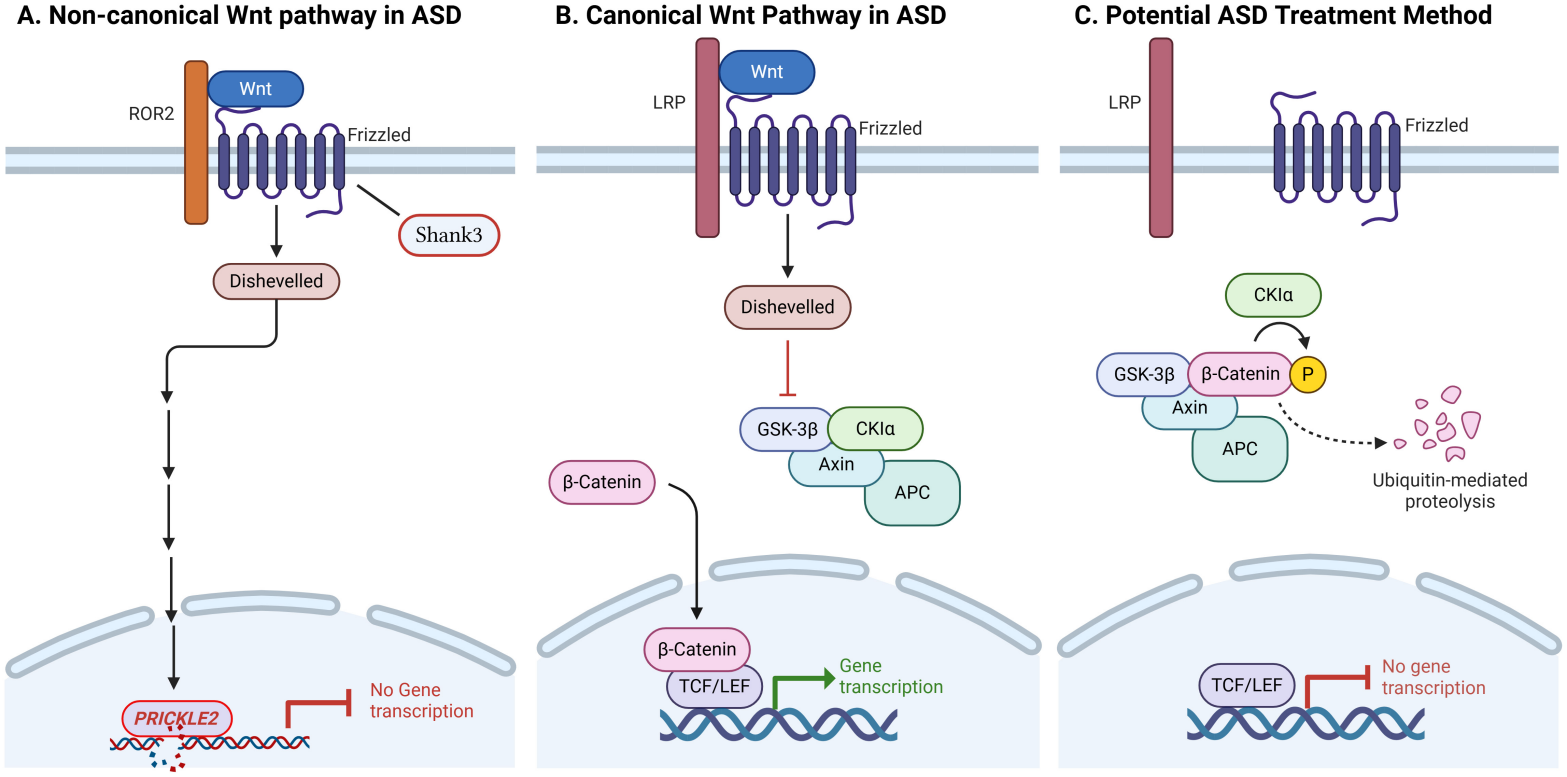
Anomalies in the canonical and non-canonical Wnt signaling pathways are the cause of autism spectrum disorders (ASDs). **(A)** Disruptions in non-canonical Wnt genes, such as PRICKLE2, may exacerbate the synaptic defects that underlie ASDs. Mice with PRICKLE2 disruption exhibit atypical behavior, such as impaired social interaction, improper learning, and rigid conduct. In mouse hippocampal neurons, PRICKLE2 disruption resulted in decreases in post-synaptic density size, synapse number, and dendritic branching. **(B)** The canonical Wnt pathway is β-catenin-mediated. Wnt target genes associated with ASD can be activated by stabilized β-catenin entering the nucleus and dislodging Goucho/transducin-like enhancer of split (TLE) repressors from T-cell factor/lymphoid enhancer factor (TCF/LEF) transcription factors. **(C)** The destruction complex, which includes Axin, adenomatous polyposis coli (APC), casein kinase 1 alpha (CK1α), and glycogen synthase kinase 3 beta (GSK3β), constitutively breaks down β-catenin in the absence of Wnt activation or due to the presence of an inhibitor. The breakdown of β-catenin through the ubiquitin-mediated proteasome pathway is facilitated by phosphorylation by GSK3β and CK1α. Deactivation of Wnt could be used as a potential ASD treatment method.

There are several phases of cerebellar development when Wnt signaling pathways are implicated. Specifically, Wnt1 contributes to the preservation of the midbrain-hindbrain area during the early stages of cerebellar anlage formation (Brault et al., 2001). E12.5 also expresses Wnt1 from migratory granule cell progenitors (Shimamura et al., 1994). Wnt7a, which enhances afferent mossy fiber synapses, is also expressed by mature granule cells (Hall et al., 2000). Disruptions in non-canonical Wnt genes, such as PRICKLE2 may exacerbate the synaptic defects that underlie ASDs. ASD patients' PRICKLE2 variations result in a loss of PRICKLE2 protein function, and may be a contributing factor to neurological dysfunction in disorders like ASD (Sowers et al., 2013; Figure 2A). The WNT “off state” is triggered, and the cytosolic β-catenin is attached to its destruction complex, which is made up of CK-1, APC, AXIN, and GSK-3β. Following CK1's phosphorylation of the Ser45 residue, GSK-3β phosphorylates β-catenin on the Thr41, Ser37, and Ser33 residues. The proteasome then breaks down phosphorylated β-catenin. Thus, in the absence of WNT ligands, the cytosolic level of β-catenin is maintained at a low level. The TCF/LEF complex cannot activate the target genes if β-catenin is not translocated to the nucleus (Figures 2B, C).

An elevated JAK/STAT signaling induction is observed in ASD. According to preliminary data, neurodevelopmental problems are associated with the JAK/STAT pathway (Mount et al., 2007). The JAK/STAT pathway is essential for the survival, proliferation, and differentiation of various cell types (Adamson et al., 2009). According to research, STAT5 is crucial for the development of cortical interneurons in the developing brain (Markham et al., 2007). Significantly, target modulators have been shown to reduce neuronal issues linked to both acute and long-term neurological impairments. Modulators of JAK-STAT, mTOR, and PPARγ signaling have therapeutic significance against neuronal dysfunctions (Kumar et al., 2023).

PPAR-gamma agonists could be used along with JAK-STAT inhibitors as target therapeutic interventions for autism. There is involvement and mutual regulation of JAK-STAT and PPAR-gamma signaling in controlling multiple pathological factors associated with autism (Khera et al., 2022). ASD pathologies may be lessened by enhancing cytokine and JAK/STAT signaling. mTOR-dependent increased spine density is associated with ASD-like stereotypies and cortico-striatal hyperconnectivity in Tsc2 haplo-insufficient mice (Pagani et al., 2021). An analogous cortical-striatal hyperconnectivity is observed in children with idiopathic ASD, which can be used as a connectivity fingerprint for ASD-dysregulated genes interacting with mTOR or Tsc2 (Pagani et al., 2021). The mTOR and the MAPK pathways showed increased activity, which are primary regulators of protein synthesis and synaptogenesis. Based on their clinical diagnosis, rpS6, p-eIF4E, TSC1, and p-MNK1 expression levels varied, inferring that various components of signaling pathways form a molecular signature for the severity of autism (Rosina et al., 2019). Loss of CHD8-mediated regulatory control may perturb normal proliferation and differentiation of neuronal progenitors, given the functions of the genes strongly affected by CHD8 knockdown in human neural stem cells (hNSCs), which may result in altered numbers or relative proportions of neuronal populations derived later in cortical development (Cotney et al., 2015). Transcriptional and splicing dysregulation are the underlying mechanisms of neuronal dysfunction that provide strong evidence for convergent molecular abnormalities in ASD (Voineagu et al., 2011; Yizhar et al., 2011).

#### Epigenetics of ASD

Investigating epigenetic pathways in idiopathic autism is intriguing not only because it provides insight into higher-order regulation of gene expression but also because exposure to biological modulators and environmental variables might impact these alterations. Therefore, the relationship between genotype and extrinsic (environmental) or intrinsic (biological) factors that contribute to ASDs may be mediated by epigenetics (Nguyen et al., 2010). DNA methylation is identified as a potential mechanism for epigenetic regulation in idiopathic autism by global methylation profiling of lymphoblastoid cell lines from phenotypically discordant monozygotic twins and non-autistic siblings (Nguyen et al., 2010). Key targets, such as increased levels of chemokine receptors and ZIC family genes, have been identified by recent studies on autism spectrum disorder (ASD).

## Nuclear hormone receptors

Nuclear hormone receptors (NHRs) are transcription factors that interact with co-repressors and co-activators to control the expression of various genes. A combination of functional and computational research has established the evolutionary alterations of NR1H and NR1I receptors across vertebrates and their potential divergence from ancestral receptors that initially appeared in invertebrates (Krasowski et al., 2011). Several studies have linked defects in NHRs to autism in humans (Kong et al., 2020; Olivares et al., 2016). To date, the human genome has been identified as having 48 NHRs, while the mouse genome has 49 (Zhang et al., 2004). Nuclear receptors play important roles in reproduction, development, and physiology by controlling gene expression. This, in turn, affects the growth (Fu et al., 2019), differentiation (Ma et al., 2022), and death (Tschuck et al., 2023; Lambrecht et al., 2023) of various cell types. Specific roles for NHRs linked to ASD are described below.

### NHR structure and regulation

NHRs are built with conserved domain organization, typically including a hinge region, a ligand-binding domain (LBD) with a second transactivation domain, a variable amino-terminal domain (A/B), and a DNA-binding domain (DBD) with zinc finger motifs (De Bosscher et al., 2020; Pavek, 2016; Lavery and McEwan, 2005). As NHRs are natural ligand-activated transcription factors, endogenous substances such as bile acids, retinoids, steroid hormones, thyroid hormones, and vitamin D act as their ligands (Tao et al., 2020). PXR and CAR are examples of these promiscuous receptors for the structurally diverse class of ligands that function as agonists (Bwayi et al., 2022). When ligands bind to specific hormone response regions on DNA, they cause changes in shape that enable nuclear translocation, dimerization, and the regulation of gene expression (Li et al., 2019). NHRs can control gene expression by directly binding to target genes and interacting with other nuclear receptors (Aranda and Pascual, 2001; Ogawa et al., 2005; Béchet, 1980; Huggins and Greene, 2023; Jiang et al., 2024; Ratman et al., 2016; Wang et al., 2023; Miao et al., 2019).

### Role of NHRs in ASD

Because of their important roles in regulating transcription, brain development, controlling gene expression, and their connection to known risk factors for ASD, NHRs are increasingly recognized as key target genes in ASD. Histone deacetylases (HDACs) change chromatin structure and gene expression. They are linked to nuclear receptor corepressors (NCORs), which play a vital role as transcriptional co-regulators. The NCOR complex connects gene-environment interactions and changes in gene regulation in the context of ASD by interacting with well-known ASD-related genes like MECP2 and TBL1XR1 (Mastrototaro et al., 2021). For instance, Rett syndrome, is associated with mutations that disrupt MECP2-NCOR interactions (Lyst et al., 2013). Prenatal exposure to HDAC inhibitors like valproic acid (VPA), which is an environmental risk factor for ASD, interferes with NHR-related mechanisms of gene repression (Kong et al., 2020). Genes linked to neurotransmitter systems that are disrupted in ASD include glutamate receptors (NMDA and mGluR receptors; Lee et al., 2025), genes from the GABAergic system (Zhao et al., 2022), and synaptic scaffolding (SHANK3; Mihalj et al., 2024), are affected by NHRs. Key features of ASD are the imbalance between excitatory and inhibitory signals and problems with synapses, which worsen due to the disruption of these genes caused by faulty NHR corepressor activity. Like in other ASD models, mouse models that lack NCOR components show problems with excitability, changes in glutamate receptor expression, and issues with cognitive and social functions (Lim et al., 2022; Kong et al., 2020). Hormones and vitamins activate NHRs (Krasowski et al., 2011), which are ligand-dependent transcription factors that control gene networks important for synapse formation, brain growth, and neuronal differentiation (Olivares et al., 2016). They also change epigenetic markers that are crucial for maintaining a stable gene expression during development. For instance, members of the NHR family, such as retinoic acid receptors and thyroid hormone receptors, influence the maturation of brain circuits connected to social behaviors and cognitive impairment in ASD (Olivares et al., 2016; Kong et al., 2020). NHRs play an important role in controlling cell fate, hence 15–20% of all pharmaceutical medications target them (Kenda and Sollner Dolenc, 2020; Pal et al., 2023). Tiny compounds like synthetic ligands or HDAC inhibitors can change their activity, making NHRs appealing targets for therapy (De Bosscher et al., 2020; Pal et al., 2023). Specific NR-targeting drugs are often used to treat various diseases. For example, raloxifene and tamoxifen target the estrogen receptor (ER), and are used to treat breast cancer and osteoporosis.

We may be able to restore disrupted transcriptional and epigenetic patterns in ASD by focusing on NHR pathways. For example, in mouse models of ASD linked to failures in NHR-related genes, researchers have shown that modifying histone acetylation with HDAC inhibitors leads to behavioral recovery (Dash et al., 2009). Additionally, given the neuroinflammatory factors of ASD, the anti-inflammatory roles of specific NHRs, such as Retinoic acid-related orphan receptor-alpha (RORA) and peroxisome proliferator-activated receptors (PPARs), may present new treatment options (Jiang et al., 2022).

### Retinoic acid-related orphan receptor-alpha (RORA)

ROR alpha 1 belongs to the unique NR1 subfamily of monomeric orphan nuclear receptors. These receptors use a variety of ways to accomplish high-affinity and selective DNA binding and hence can act as master transcription regulators (Giguère et al., 1995). RORA activates transcription from only a subset of sites to which it binds strongly as a monomer (Harding et al., 1997). RORA also selectively binds as a homodimer to a direct repeat of this monomer site with a 2-bp spacing between the AGGTCA sequences (RevDR2 site) and is a much more potent transcriptional activator on this site than on monomer sites or other direct repeats (Harding et al., 1997). Mutational analysis revealed that RORA contains both transcriptional activation and transcriptional repression domains, with the repression domain being more active in some cell types (Harding et al., 1997). The abilities of RORA polypeptides to repress transcription correlate with their abilities to interact with the nuclear receptor corepressors N-CoR and SMRT *in vitro* (Harding et al., 1997). Transcriptional regulation by RORA is complex and likely to be regulated in a cell-type and target gene-specific manner (Harding et al., 1997). RORα polymorphisms (rs11639084 and rs4774388) have been associated with ASD risk (Sayad et al., 2017). Studying twins where one sibling has autism and the other does not, revealed increased CpG island methylation at the upstream RORα promoter sites of the twin with ASD (Nguyen et al., 2010). Global methylation profiling revealed that the RORα protein levels were significantly reduced in the brains of individuals with ASD due to epigenetic alterations at the RORα gene (Nguyen et al., 2010). Experimental correlation of methylation with gene expression data revealed that RORA exhibited methylation-specific gene silencing in conjunction with enhanced methylation of particular CpG sites in corresponding upstream regulatory CpG islands (Nguyen et al., 2010). Perhaps likely not all populations carry methylation of the RORA promoter region as an epigenetic risk factor for autism (Salehi et al., 2017). Post-mortem examination of age-matched case-control individuals also showed decreased expression of RORα protein in the prefrontal cortex and the cerebellum of autistic individuals (Hu et al., 2009). These findings are significant because studies on the RORαsg mice indicate that RORα protein is involved in several processes relevant to autism including Purkinje cell differentiation (Hadj-Sahraoui et al., 2001; Boukhtouche et al., 2006b; Chen et al., 2013), cerebellar development (Gold et al., 2003; Harding et al., 1997), brain lipid homeostasis (Chen et al., 2020), protection against oxidative stress and inflammation (Boukhtouche et al., 2006c; Delerive et al., 2001; Nejati Moharrami et al., 2018; Yue et al., 2020), and the circadian rhythm (Fatemi et al., 2012).

#### Role of RORA in CNS development

During CNS development and in mature neurons, modifications in the dendritic architecture refine the function of neural circuits (Bottjer and Arnold, 1997). RORA was shown to regulate dendritic remodeling during CNS development (Boukhtouche et al., 2006c). The deficiency of Purkinje cells is a consistently identified neuroanatomical abnormality in the brains of individuals with ASD (Palmen et al., 2004; Polleux and Lauder, 2004) and RORα is critical in the development of Purkinje cells (Hadj-Sahraoui et al., 2001; Boukhtouche et al., 2006a; Doulazmi et al., 1999; Hamilton et al., 1996). Purkinje cells inhibit the action potentials from deep cerebellar nuclei and vestibular neurons in the cerebellum. By regulating the rate at which signals fire in the cerebellum, Purkinje cells produce precise motor coordination. Disruption of Purkinje function causes multiple motor defects associated with ASD. Purkinje cells express RORα very early in development which continues during adulthood (Ino, 2004). In RORαsg mice, most of the Purkinje cells die within the first month of life (Herrup and Mullen, 1979; Dussault et al., 1998; Vogel et al., 2000; Doulazmi et al., 2001). The surviving Purkinje cells fail to mature and develop spiny branchlets (Landis and Sidman, 1978; Sotelo, 1978). RORα is necessary for the retraction of transient dendrites in the early development of Purkinje cells to establish a mature dendritic tree, an essential step in Purkinje cell maturation (Boukhtouche et al., 2006a). RORα deficiency in adult mice (Chen et al., 2013) also creates defects in Purkinje cells such as premature dendritic atrophy and death (Hadj-Sahraoui et al., 2001), Therefore RORα is a terminal differentiation gene that defines the functional properties of a mature Purkinje cell from development to maintenance, throughout its life. The genetic programs in developing Purkinje cells, analyzed daily during mouse prenatal development revealed that RORα bound to the promoter sites and controlled the expression of Shh, Slc1a6, Itpr1, Pcp4, and Pcp2 (Gold et al., 2003). These RORα target genes provide mitogenic drive and are also required for reciprocal signals between Purkinje, granule, and molecular cells in cerebellar development. Studies in RORαsg mice suggest a possible role of RORα in the expression of cell proliferation, neuronal differentiation, and mature neuron markers (Ki67, DCX, and NeuN, respectively) in the dentate gyrus (Yi et al., 2010). The dentate gyrus is the first region where all sensory modalities converge to form unique representations that bind the different sensory stimuli together, thereby playing a critical role in learning and memory. Liver X-receptor (LXR)β, a nuclear receptor closely related to RORα has been linked to dentate gyrus development abnormalities and autism spectrum disorders (Cai et al., 2018). Exogenous RORα expression in RORαsg mice partially restored the normal cerebellum Purkinje cell count and neuronal architecture (Iizuka et al., 2016). The RORA-related neurodevelopmental disorder triad comprises developmental retardation, cerebellar features, and a spectrum of myoclonic epilepsy (Talarico et al., 2025). In a study, out of 40 individuals carrying RORA pathogenic/likely pathogenic variants collected through an international collaboration, 32/40 presented with developmental delay, 25/34 with cerebellar signs and 22/32 with intellectual disability. Cerebellar symptoms were divided into early-onset, late-onset, and progressive subgroups. Cerebellar hypoplasia, atrophy, or both (16/25) were more frequent in individuals with missense variants in the DNA-binding domain. Epilepsy (18/38), with prominent myoclonic seizure types (11/18), was classified in Nationa Library of Medicine (2024) genetic generalized epilepsy (10/18); National Institute of Mental Health (2022) developmental and epileptic encephalopathy (5/18) and (3) unclassified (*n* = 3/18). A participant with rapid deterioration of visual acuity and cone/rod dystrophy was also reported (Talarico et al., 2025). Furthermore, RORA mutations cause “Intellectual developmental disorder with or without epilepsy or cerebellar ataxia” syndrome (IDDECA). While *de novo* dominant toxic variants cause intellectual disability, ataxia, and cerebellar atrophy, *de novo* or dominantly inherited loss-of-function variants cause intellectual disability with autistic features. These two different phenotypes show what kind of functional effect the variant has (Guissart et al., 2018). Twenty-five of the 53 RORA gene variants found so far are classified as pathogenic or possibly pathogenic whose allelic frequencies range from low to very low (Leiden Open Variation, n.d.).

#### RORA and neuronal gene regulation

**Figure 3:**
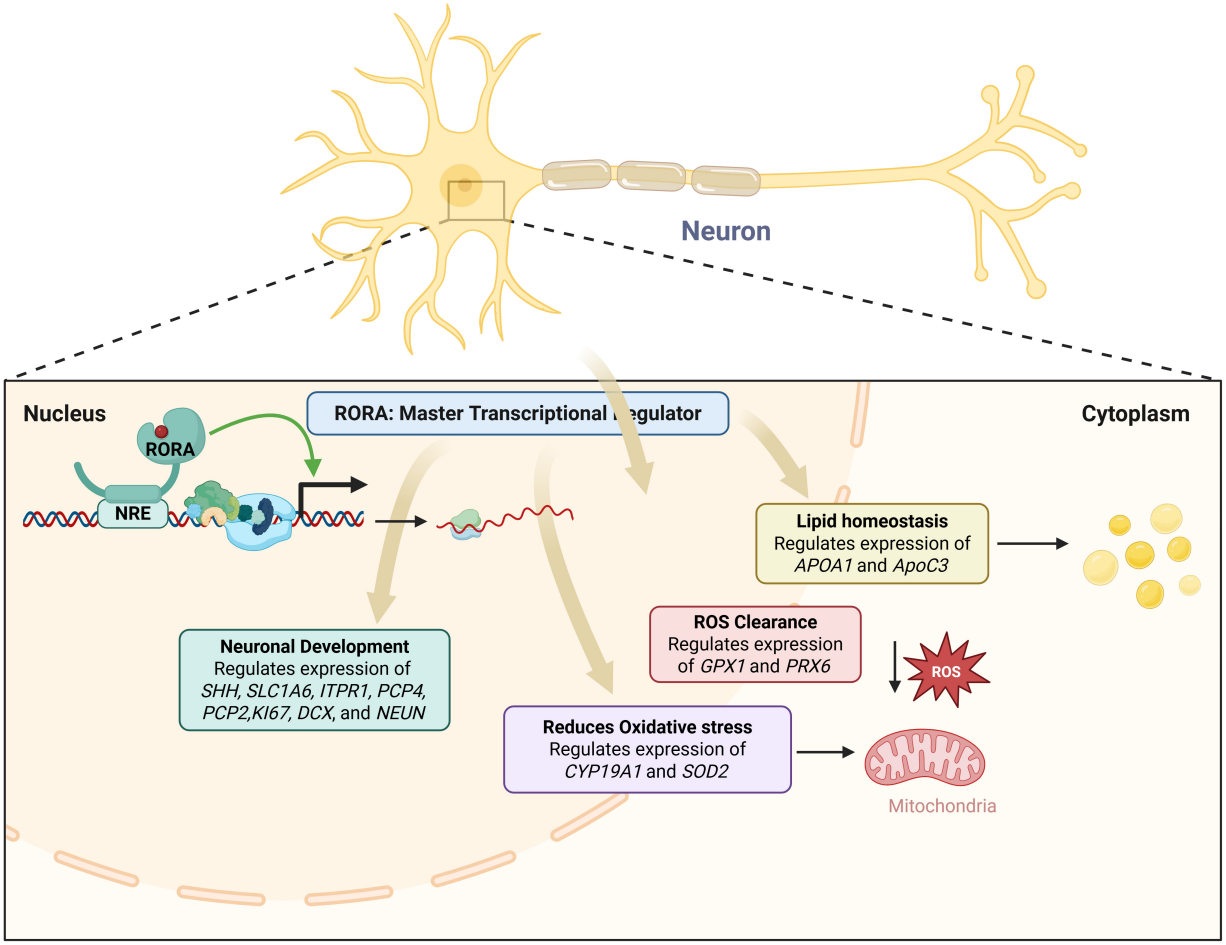
An overview of RORA in regulation of the pathways implicated in ASD.

Multiple genes associated with ASD are direct RORα targets and reduction of RORα expression results in reduced expression of these genes leading to ASD (Sarachana and Hu, 2013). Individuals with ASD show significant disruptions in their circadian cycles (Hu et al., 2009; Bourgeron, 2007; Melke et al., 2008; Nicholas et al., 2007), and RORα plays a critical role in the regulation of the circadian rhythm (Jetten, 2009). Environmental and metabolic factors of ASD have also been reported to affect RORα expression levels (Kanlayaprasit et al., 2021; Xiao et al., 2022; Yu et al., 2022). Sex differences in the expression of RORα and its target genes in the brain have been investigated as a potential contributor to the sex bias in autism (Hu et al., 2015). A strong correlation between RORα and aromatase levels was observed in males with ASD but not in females, suggesting that females might have compensatory mechanisms to offset RORα deficiency. Additionally, the RORαsg mice display behaviors associated with autism including abnormal spatial learning, reduced exploration, limited maze patrolling, and increased perseverative behavior relative to WT mice (Lalonde and Strazielle, 2008; Goodall and Gheusi, 1987; Lalonde, 1987; Lalonde et al., 1987, 1988). This is especially true when examining dysregulated genes with systemic functions like apoptosis and circadian rhythm (Nguyen et al., 2010). By augmenting the generation of the antioxidant proteins glutathione peroxidase 1 and peroxiredoxin 6, the overexpression of human RORA1 offers neuroprotection by reducing the buildup of reactive oxygen species (ROS) brought on by stress (Boukhtouche et al., 2006c; Figure 3). Glutathione peroxidase 1 and peroxiredoxin 6 are the main mediators of RORA's neuroprotective action (Boukhtouche et al., 2006c; Figure 3). Gpx1 and Prx6, two crucial enzymes in ROS clearance, were shown to be overexpressed in hRORa1-overexpressing cells by a gene candidate analysis employing real-time RT-PCR (Boukhtouche et al., 2006c). Through its ability to degrade hydrogen peroxide, the enzyme primarily responsible for peroxide detoxification in mammalian cells is encoded by the Gpx1 gene (Boukhtouche et al., 2006c). It has been demonstrated that its overexpression via viral vector infection protects against cell death brought on by experimental stroke *in vivo* (Hoehn et al., 2003). T regulatory cells that reside in tissues like the gut and express RORA may be able to reduce allergic inflammation in organs other than the skin (Schiering et al., 2014; Figure 3). RORA expression was noticeably higher in human skin T regulatory cells than in blood T regulatory cells, indicating that the results are relevant to humans (Malhotra et al., 2018; Figure 3). A positive circuit for hypoxia signaling is activated when ROR-alpha and its ligands activate HIF-1-alpha (Kim et al., 2008; Figure 3). RORα is a key regulator of TH17 cell growth, autoimmune, and chronic inflammation, as shown by a mix of genetic, molecular biology, and pharmacological methods (Wang et al., 2021). By controlling oxidative stress, cellular quality control procedures, and cell death mechanisms, RORs play important protective roles in the onset and progression of several human diseases (Nematisouldaragh et al., 2024). Given that it induces cerebellar developmental abnormalities that impact downstream targets and regulate extracerebral systems like inflammation, circadian rhythms, and sexual dimorphism, RORα deficiency is probably a major contributor to neuro-developmental disorders (Ribeiro and Sherrard, 2023). Similar to the androgen receptor complex that is thought to develop on the promoter and enhancer of the prostate-specific antigen gene, RORα-dependent complexes can form at independent locations or communicate between proximal and distal sites by looping (Shang et al., 2002). The level of expression of some genes relates directly to the severity of the phenotype and may serve as useful candidates for eQTL analyses (Hu et al., 2009). Network analysis of genes that are shared between the severely language-impaired and mild ASD subgroups reveals a set of genes that are probably critical concerning the neurological and metabolic abnormalities of ASD (Hu et al., 2009). A set of differentially expressed genes, most of which lie in non-coding regions, are shared among all three ASD subgroups examined (Hu et al., 2009). Differences between affected functions and pathways among the different phenotypic groups may be responsible for the differences in symptom severity observed in autism (Hu et al., 2009). Sonic Hedgehog is a direct transcriptional target of RORα based on the array data's decreased expression of multiple EGL-expressed cell cycle and proliferation marker genes. By stimulating Amyc, its binding partner Baf53, and different cyclins (Kenney et al., 2003; Kenney and Rowitch, 2000), Purkinje cell-derived SHH has been demonstrated to be both sufficient to induce granule precursor proliferation in postnatal explant cultures in a dose-responsive manner and required for normal levels of granule cell genesis (Wechsler-Reya and Scott, 1999). While various nuclear receptors' cofactors can interact with RORα *in vitro* (Atkins et al., 1999; Delerive et al., 2002; Lau et al., 1999), particular DNA factors that are recruited to the promoters of RORα target genes in a manner that is dependent on RORα. Tip60, SRC-1, and β-catenin are among the factors that RORα recruits and play a functional role in RORα-dependent transcriptional activation. It has recently been demonstrated that Tip60 recruitment is a necessary coactivator for particular NF-κB gene targets (Baek et al., 2002). Tip60 is recruited to each of three RORα-responsive promoters in a manner dependent on RORα. Coactivators are recruited to target gene promoters uniquely and distinctly, that is, dependent on RORα, and numerous recruited cofactors exhibit functional activity. The calmodulin inhibitor Pcp4, the IP3 receptor and calmodulin target Itpr1, the Itpr1 binding partner Cals1, and the calcium buffer Calb1 are among the calcium signal transduction genes that exhibit RORα-dependent expression. The array data's time course indicates that these genes' expression is drastically decreased by E17.5, before Purkinje cell number loss (Vogel et al., 2000). This suggests that the variations in expression are due to a modified regulatory mechanism rather than subsequent disease. RORA1 and NGFI-B utilize distinct subdomains of the DBD carboxy-terminal extension and therefore reveal a novel strategy by which monomeric nuclear receptors recognize their cognate HREs. Male and female hormones differentially regulate the expression of a novel autism candidate gene, retinoic acid-related orphan receptor-alpha (RORA), in a neuronal cell line, SH-SY5Y (Sarachana et al., 2011). RORA transcriptionally regulates aromatase, an enzyme that converts testosterone to estrogen (Sarachana et al., 2011). Aromatase protein is significantly reduced in the frontal cortex of autistic subjects relative to sex- and age-matched controls and is strongly correlated with RORA protein levels in the brain (Sarachana et al., 2011). RORA has the potential to be under both negative and positive feedback regulation by male and female hormones, respectively, through one of its transcriptional targets, aromatase, suggesting a mechanism for introducing sex bias in autism (Sarachana et al., 2011).

#### RORA's regulation of brain lipid metabolism

**Figure 4:**
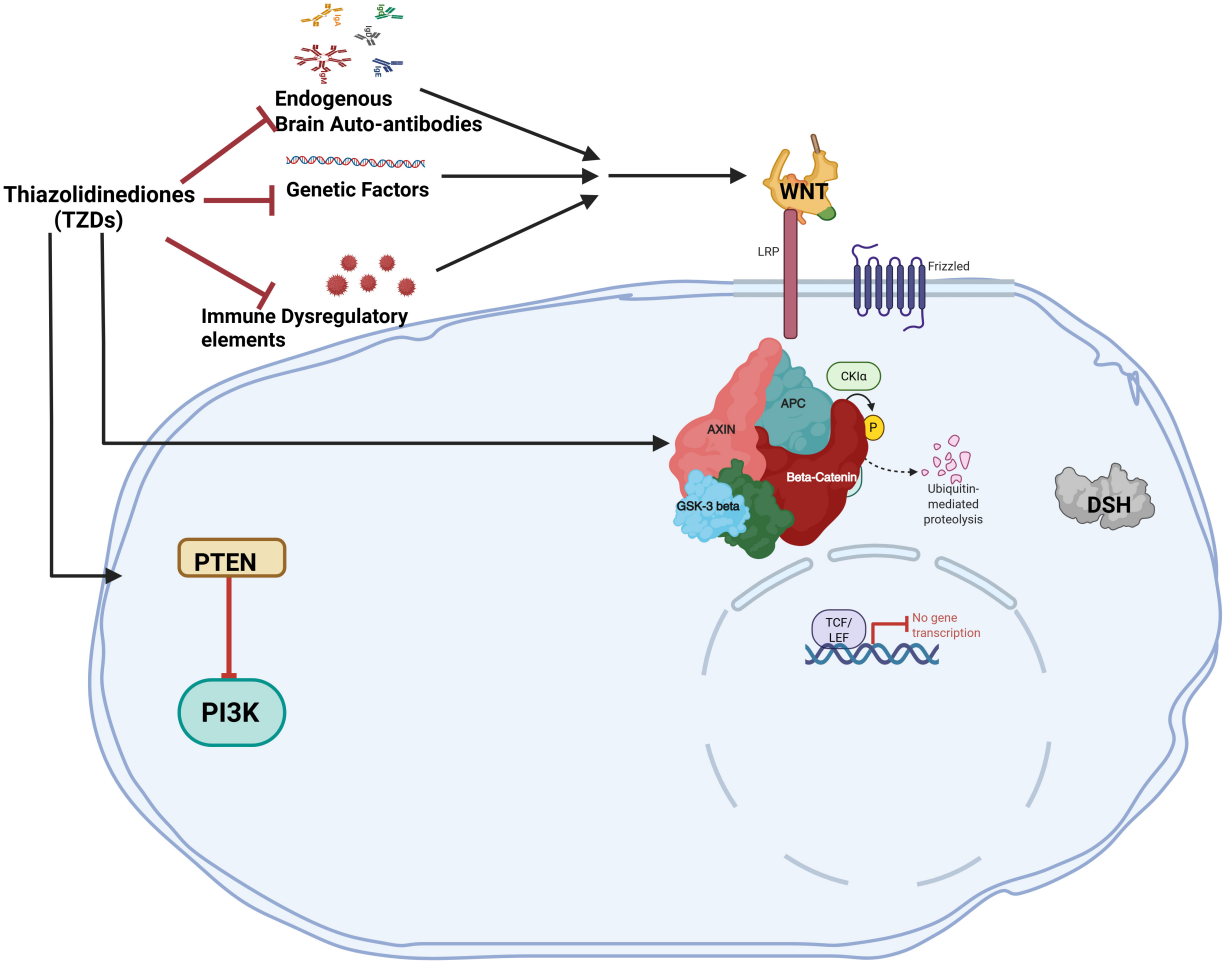
The canonical WNT/β-catenin pathway and PPARγ have an antagonistic relationship in ASD. By preventing the release of inflammatory factors and cytokines. Thiazolidinediones act as PPARγ agonists which can directly prevent neuroinflammation. PPARγ agonists directly activate DKK1 to block the WNT pathway. When PPARγ agonists are administered, the WNT pathway is downregulated due to the antagonistic relationship between PPARγ and the canonical WNT/β-catenin pathway.

Polyunsaturated fatty acids (PUFA), such as omega-6 and omega-3 fatty acids, are critical for early brain development (Crawford et al., 2003; Haag, 2003; Lauritzen et al., 2001; Martinez, 1992). The predominant PUFA species, arachidonic acid (n-6) and docosahexaenoic acid (DHA; n-3; Brenna and Diau, 2007; Diau et al., 2005), are enriched for neuronal growth, synaptogenesis, neuronal survival, and modulation of neurotransmitters (Darios and Davletov, 2006; Kim and Spector, 2018; Kuperstein et al., 2008). Although abnormal neural lipid metabolism in individuals with ASD has not been extensively investigated, abnormal lipid metabolism has been reported as one of the plasma biomarkers (Das, 2013; Tamiji and Crawford, 2010) and PUFA interventions in animal models may alleviate autistic-like cognitive and social behaviors (Weiser et al., 2016; Shultz et al., 2008, 2009). RORα regulates lipoprotein homeostasis (Vu-Dac et al., 1997) and RORαsg mice exhibit aberrant lipid metabolism (reduced serum cholesterol and triglycerides) due to reduced expressions of ApoA1 and ApoC3, respectively (Raspé et al., 2001; Mamontova et al., 1998). RORα may regulate lipogenesis and mitochondrial fatty acid oxidation by suppressing expressions of peroxisome proliferator-activated receptor-γ (PPARγ), its co-activator PGC1α and lipin1 (Lau et al., 2008; Kim K. et al., 2017). Recent reports suggest that RORα deficiency delays all fatty acid accretions during critical periods of brain development, however, deficiency in the omega-3 PUFA species—DHA, persists in adult RORαsg mice and is not rescued by dietary DHA supplementation (Chen et al., 2020). Similarly, a meta-analysis of case-control cohorts found selectively lower DHA levels in the blood of ASD children (≤12 years) despite no differences in reported dietary intakes with the control group (Mazahery et al., 2017). Although DHA supplementation reversed some impairments in mouse models of ASD (BTBR and serotonin transporter knockout; Matsui et al., 2018; van Elst et al., 2019), human trials reporting an increase in blood DHA after dietary supplementation failed to observe improvements of social behavior in children with ASD (Bent et al., 2011; Voigt et al., 2014). This provides additional evidence that RORα deficiency may affect the efficacy of dietary DHA supplementation either by slowing DHA incorporation into, and/or accelerating loss of DHA from brain phospholipids. Therefore, in humans, RORα upregulation by gene therapy could be a viable option for individuals with ASD (Figure 4) in combination with supplements and other medical options for symptom management.

#### RORA protects neurons from oxidative stress

Oxidative stress is a common feature in autism cases, which may be further exacerbated due to the presence of genetically susceptible alleles (Chauhan and Chauhan, 2006, 2009; Deth et al., 2008; Kern and Jones, 2006; Wells et al., 2009). Limited antioxidant capacity, high energy requirement, and high levels of iron and PUFA in the brain increase its vulnerability to oxidative stress. Postmortem studies on brain tissues of individuals with ASD have shown elevated levels of oxidative damage and reduced antioxidant capacity as compared to age-matched control subjects. Individuals with ASD show higher levels of metabolites such as lipid hydroperoxide (from fatty acid oxidation; Chauhan et al., 2011), malonyl dialdehyde (from lipid peroxidation; Chauhan et al., 2012), 8-hydroxy-2′-deoxyguanosine (from oxidative DNA damage; Chauhan et al., 2010; Sajdel-Sulkowska et al., 2009), protein carbonyl (from protein oxidation; Chauhan et al., 2010), 3-nitrotyrosine (from protein nitration; Sajdel-Sulkowska et al., 2008), carboxyethyl pyrrole (a lipid-derived oxidative protein modification; Evans et al., 2008). Overexpression of human RORα1 in cultured mouse cortical neurons increases the expression of the antioxidant proteins glutathione peroxidase 1 (Gpx1) and peroxiredoxin 6 (Prx6), decreases the levels of reactive oxygen species (OS; Figure 4), and protects neurons from apoptosis due to oxidative stressors such as β-amyloid peptide, c_2_-ceramide, and H_2_O_2_ (Boukhtouche et al., 2006c). Another study reported that maternal diabetes in mice induced oxidative stress in the brains of their offspring and led to autism-like behavior (Pagani et al., 2021). Both the oxidative stress and ALB in the mice offspring were accompanied by downregulation of RORα and its target genes CYP19A1 (aromatase) and Sod2 (superoxide dismutase). Post-natal overexpression of RORα in the offspring rescued both ALB and neuronal oxidative stress, while sh-RNA knockdown had the reverse effect and worsened the ALB (Yu et al., 2022).

#### RORA protects neurons from neuroinflammation

A striking feature common to individuals with ASD is the presence of ongoing neuroinflammation over a broad age range (Vargas et al., 2005) with elevated levels of cytokines and chemokines such as IL-6, TGFβ1, TNFα, CCL2, and CCL17 in the cerebellum and other areas of the brain (Vargas et al., 2005; Chez et al., 2007; Li et al., 2009; Wei et al., 2011). Transcriptome organization patterns in the brain of individuals with ASD show abnormalities in gene co-expression networks associated with immune activation (Voineagu et al., 2011). Disrupted monocyte/macrophage function under resting conditions is reported in ASD (Ashwood et al., 2009; Enstrom et al., 2010) with decreased production of regulatory (anti-inflammatory) cytokines TGFβ1 and IL-10 (Ashwood et al., 2004, 2008), and elevated levels of antibodies against cerebellar proteins (Goines et al., 2011), all of which are associated with worsening behavioral phenotype. Astrocytes are multifunctional macroglial cells that provide neurons with structural and metabolic support (Volterra and Meldolesi, 2005; Marty et al., 2005), absorb neurotransmitters, modulate ion concentration, and synaptic transmission (Haydon and Carmignoto, 2006; Parpura and Haydon, 2000; Fellin et al., 2006). They also maintain the blood-brain barrier (Parri and Crunelli, 2003), act as chemosensors (McDougal et al., 2011), promote myelination (Ishibashi et al., 2006), axon regeneration (Anderson et al., 2016), and drive the molecular oscillations in the circadian clock (Brancaccio et al., 2019). Astrocytes' role in neuroinflammation is becoming apparent (Volterra and Meldolesi, 2005; Song et al., 2002; Farina et al., 2007). As effectors of innate immunity in the brain, astrocytes are principally activated by the NF-κB signaling pathway and produce high levels of IL-6 (Van Wagoner and Benveniste, 1999) by a RORα-dependent mechanism (Delerive et al., 2001; Journiac et al., 2009). Although astrocytes from RORαsg mice were reported to have lower resting IL-6 levels than WT mice, upon stimulation by pro-inflammatory cytokines IL-1β and TNFα, the IL-6 levels were significantly higher in RORαsg astrocytes indicative of a pro-neuroinflammatory drive in the absence of RORα (Journiac et al., 2009).

## Peroxisome proliferator-activated receptor gamma (PPARγ)

Studies demonstrated an involvement and mutual regulation of PPAR-gamma and JAK-STAT signaling in controlling multiple pathological factors associated with autism (Khera et al., 2022). A direct repeat (DR) of a hexanucleotide sequence [5′-TGA (A/C/T) CT] divided by a single nucleotide (DR1) makes up the binding motif of the known PPREs. Several other nuclear receptor response elements, including the TR, RAR, VDR, COUP transcriptional factor, and retinoid X receptor (RXR), also contain this type of sequence (Umesono et al., 1991; Yu et al., 1991). The positioning and spacing of the half-site motifs allow these receptors to differentiate between different target elements even though they may all determine the identical half-site motif. Diverse receptors that can operate either positively or negatively control a target gene's expression may also target the same location. For example, retinoid receptors and homomeric and heteromeric complexes of the transcription factors RXR and COUP detect the DR1 (Kliewer et al., 1992b,a). Additionally, it has recently been established that for the TR, RAR, and VDR to bind to their respective target DNA sequences effectively, they all need an auxiliary factor (RXR; Yu et al., 1991; Kliewer et al., 1992a,b; Bugge et al., 1992; Leid et al., 1992). In particular, PPARγ: (1) causes adipogenesis in healthy SAT but not in unhealthy VAT (Mayerson et al., 2002; Miyazaki et al., 2002; Adams et al., 1997); (2) increases the expression of uncoupling protein 1 in BAT (Cao et al., 2004; Zhang et al., 2014); (3) raises serum adiponectin levels (Hirose et al., 2002); (4) inhibits inflammation (Pascual et al., 2005); and (5) encourages mitochondrial biogenesis and lowers reactive oxygen species production in the mitochondria (Fujisawa et al., 2009). Although TZDs are complete agonists of PPARγ, as was previously mentioned, they have unfavorable side effects that limit or prohibit their clinical use. A new generation of agonists with improved safety profiles and wider applicability may be developed due to a growing understanding of PPARγ signaling.

Two different signaling pathways are triggered when TZDs and other ligands directly bind to PPARγ. Through the recruitment of co-activators like PPARγ co-activator-1α (PGC-1α), PPARγ dimerization with the retinoid X receptor (RXR; Spiegelman, 1998), agonist-dependent conformational changes in the activation function 2 (AF-2) domain of PPARγ, and the binding of this complex to peroxisome proliferator response elements (PPREs) in the promoters of target genes, cis-activation drives transcription. Alternatively, it has been established that ligand-bound PPARγ decreases inflammation by a process known as trans-repression. The PPARγ C126A/E127A mutant, which is unable to cis-activate but still able to repress lipopolysaccharide-induced genes, demonstrates that trans-repression is independent of PPARγ's ability to bind DNA (Li et al., 2000). RXR dimerization is also not necessary for this mode of repression; however, the small ubiquitin-like modifier must sumoylate PPARγ2 at K395 and then bind PPARγ, nuclear receptor co-repressor, and histone deacetylase to NFκB and AP-1 complexes that control the transcription of inflammatory response genes (Pascual et al., 2005).

## Other NHRs

Liver X receptors (LXRs) are important modulators of lipid and cholesterol metabolism (Kalaany and Mangelsdorf, 2006). More recently, LXRs have been demonstrated to adversely affect the Hedgehog signaling pathway, which is implicated in cancer and embryonic development (Gill et al., 2008; Kim et al., 2009). LXRα is expressed at similar levels in a wide range of tissues, which is the foundation for an alternate term for this receptor called “ubiquitous receptor” (Song et al., 1995). A study revealed that the brain's Aβ generation was reduced by LXRβ through inhibition and degradation, further triggering the expression of autophagy-related proteins by decreasing the expression of proteins related to Ras/Raf/Erkl/2 signaling pathway, thereby improving autism behaviors (Zhang et al., 2016).

## Therapeutic options for ASD

### Drug molecules under investigation

ASD related irritability can be treated by using antipsychotics such as risperidone and aripiprazole, which are approved by the US FDA (Lamy and Erickson, 2018). ASD patients have a greater risk of side effects with methylphenidate, such as depressive mood disorder symptoms, low appetite, insomnia, irritability, increased levels of social withdrawal, and lower treatment response rates compared to patients with ADHD alone (Research Units on Pediatric Psychopharmacology Autism Network, 2005; Reichow et al., 2013). Atomoxetine moderately improved ADHD related symptoms despite frequent adverse events such as fatigue, nausea, low appetite, and early morning awakening (Harfterkamp et al., 2012). Extended-release Guanfacine as an alpha-2 agonist was effective for ADHD in children with ASD (Scahill et al., 2015). Randomized, placebo-controlled trials in ASD youth showed an over 50% reduction in the irritability score when treated with Risperidone (Levine et al., 2016). Another antipsychotic drug approved by the FDA, aripiprazole, for treating irritability associated with autism showed lower severity scores on the ABC-I and the CGI-I scales, but with weight gain being a common side effect (Wink et al., 2014). Topiramate (Mazzone and Ruta, 2006) and Divalproex (Hollander et al., 2010), the anticonvulsant drugs, are effective for treating irritability associated with ASD. The repetitive behaviors in adults with ASD treated with Fluoxetine were improved (Hollander et al., 2012). Some of the over-the-counter supplements, such as exogenous melatonin in different formulations, were effective and safe in improving the sleep patterns in children with ASD (Souders et al., 2017). A study to assess whether response to the selective serotonin reuptake inhibitor (SSRI) escitalopram in the treatment of ASD was associated with symptom improvement, observed over 6 weeks of open-label treatment (Najjar et al., 2015). Cell therapies for people with ASD have shown positive outcomes in nine out of 10 clinical trials, with no significant side effects (Nabetani et al., 2023). Certain neurobehavioral abnormalities observed in Slc7a5 mutants mice could be rescued by intracerebroventricular injections administration of leucine and isoleucine in adulthood (Tărlungeanu et al., 2016).

Treatment with Oxytocin plays a prominent role in setting social behaviors just after birth. It could be a novel therapeutic approach for the treatment of neurological diseases such as autism (Meziane et al., 2015). In a cohort of 19 children with ASD, Clonidine was found effective in decreasing night awakening, sleep initiation latency, mood instability, and improving attention deficit hyperactivity and aggressiveness (Ming et al., 2008). Aripiprazole (or Memantine) combination increased the protein levels of BDNF, p-CREB, and Glt-1 gene expression with restoration of GABA/glutamate balance, attenuation of VPA-induced neurodegenerative changes and autistic-like symptoms, and improvement of cognitive performance (Zohny et al., 2023). Maternal diabetes–mediated oxidative stress is diminished due to hematopoietic stem cells transplantation with increased Sod2 expression, apart from triggering epigenetic changes in neurons, thereby impairing autism-like behavior in autism-like offspring (Zeng et al., 2022). Mannose-PEG600-lipoic acid (Man-LA), a brain-targeted H2S donor cross-linked nano micelle, interacts with glucose transporter 1 (GLUT1) in astrocytes, facilitating a gradual release of H2S that is modulated by glutathione (Hirsch and Pringsheim, 2016; Zhang et al., 2025). Man-LA alleviates symptoms of ASD, correlating with increased expression of aerobic glycolysis enzymes, elevated lactate production, and higher H2S levels, while preventing damage to hippocampal neurons under *in-vivo* conditions (Zhang et al., 2025). Man-LA tightly binds to aldehyde dehydrogenase family three member B1 (Aldh3b1) in astrocytes in *in-vitro* conditions, upregulating its expression (Zhang et al., 2025). Within the glycolytic pathway, the Aldh3b1 gene is a potential target for ASD (Zhang et al., 2025). The exogenous melatonin administration for ASD related abnormal sleep parameters is based on evidence (Rossignol and Frye, 2014). Various clinical studies for the investigation of novel drugs are stated in Table 1.

**Table 1:** Drug molecules under investigation.

**No**.	**Drug**	**Summary**	**Study URL**	**NCT No**.	**Phase**
1	STX209 (15–30 mg)	A single-site, randomized, acute dose-response study to determine if STX209 produces a dose-dependent change in MEG target parameters compared to baseline and placebo treatment.	https://clinicaltrials.gov/study/NCT02278328	NCT02278328	Early Phase 1
2	Intranasal oxytocin	Evaluates in an open-label, crossover design the comparative efficacy and safety of intranasal oxytocin and autologous umbilical cord blood for improving the functioning of children with ASD.	https://clinicaltrials.gov/study/NCT04007224	NCT04007224	Phase 1
3	Oxytocin	Observe effects of oxytocin on individuals with autism spectrum disorder	https://clinicaltrials.gov/study/NCT03183674	NCT03183674	
4	SB-121	Multiple-dose, randomized, double-blind, placebo-controlled, cross-over single-site Phase I study. Safety and tolerability, multiple measures of AD, and mechanistic biomarkers, will be assessed in order to inform later stage trials.	https://clinicaltrials.gov/study/NCT04944901	NCT04944901	
5	Minocycline	The purpose of the study is to determine if Minocycline shows initial evidence of efficacy, safety, and tolerability in youth with ASD ages 12–22 years.	https://clinicaltrials.gov/study/NCT04031755	NCT04031755	
6	Suramin	This study is designed to test the safety and efficacy of a single, intravenous dose of suramin in autism spectrum disorders.	https://clinicaltrials.gov/study/NCT02508259	NCT02508259	Phase 1| Phase 2
7	Melatonin	Oral administration of melatonin in patients with autistic disorder. To study the relation between the melatonin dose administered and its effect on severity of autistic impairments, especially in verbal communication and play.	https://clinicaltrials.gov/study/NCT01780883	NCT01780883	Phase 2
8	Sapropterin	To provide a definitive test of the hypothesis that elevating Sapropterin in the CNS will result in measurable improvements in core symptoms of autism in young individuals, under age 6 years. Double-blind, placebo-controlled 16-week intervention.	https://clinicaltrials.gov/study/NCT00850070	NCT00850070	
10	Oxytocin (8–24 IU)	To look at the effects of intranasal oxytocin on the brain in autism spectrum disorder (ASD).	https://clinicaltrials.gov/study/NCT03033784	NCT03033784	
11	Citalopram hydrobromide	This study will determine the efficacy and safety of citalopram compared to placebo in the treatment of children with autism.	https://clinicaltrials.gov/study/NCT00086645	NCT00086645	
12	Pregnenolone	To examine the tolerability and effectiveness of pregnenolone in individuals with autism. Pregnenolone is a naturally occurring steroid hormone in the brain that has been implicated in treating various psychiatric conditions.	https://clinicaltrials.gov/study/NCT02627508	NCT02627508	
13	Buspirone	To evaluate the effects of twice-daily oral buspirone on core features of autism in autistic children aged 2–6 years.	https://clinicaltrials.gov/study/NCT00873509	NCT00873509	Phase 2 | Phase 3
14	KuvanÂ^®^	An open-label extension study available only to subjects who completed an earlier double-blind, placebo-controlled study of Sapropterin in children with autism.	https://clinicaltrials.gov/study/NCT00943579	NCT00943579	
15	Galantamine	Autism severely impacts both the affected individual and family members. The study is designed to assess the efficacy of treatment with Galantamine vs. placebo in childhood/adolescent autism fulfilling DSM-IV and Autism Diagnostic Interview (ADI) criteria.	https://clinicaltrials.gov/study/NCT00252603	NCT00252603	Phase 3
16	Bumetanide	To determine if a treatment by bumetanide presents an efficiency at the level of neuronal maturation in the autism.	https://clinicaltrials.gov/study/NCT01078714	NCT01078714	
17	Aripiprazole	To investigate the efficacy and safety of aripiprazole orally administered over 8 weeks in pediatric patients with autistic disorder.	https://clinicaltrials.gov/study/NCT01617447	NCT01617447	
18	Aripiprazole	To investigate the safety and efficacy of aripiprazole orally administered over long term in subjects who complete a short-term treatment study of pediatric autistic disorder (031-11-002 study).	https://clinicaltrials.gov/study/NCT01617460	NCT01617460	
19	Fluoxetine	To assess the effect of fluoxetine orally dissolving tablets (ODT) on the repetitive behaviors core domain in children and adolescents with autistic disorder and on improvement of symptoms and the effects on daily living of the patient's family.	https://clinicaltrials.gov/study/NCT00515320	NCT00515320	

### Challenges in the current state of drug treatments for ASD

Integrating clinical and translational treatment studies, the United States has invested more than USD 110 million in ASD research networks and institutes over the past 20 years (excluding individual initiatives). Risperidone (McCracken et al., 2002), Methylphenidate (Kim S. J. et al., 2017; Joshi et al., 2020), Guanfacine (Politte et al., 2018; Scahill et al., 2015), Secretin (Levy et al., 2003; Williams et al., 2012), Aripiprazole (Hirsch and Pringsheim, 2016), plus language intervention (Williams et al., 2024), Citalopram (Wichers et al., 2019), Fluoxetine, Intranasal Oxytocin (Sikich et al., 2021), Escitalopram (Owley et al., 2010), Buspirone (Chugani et al., 2016; Ghanizadeh and Ayoobzadehshirazi, 2015), are some of the molecules which were investigated. Integrating clinical and translational treatment studies, the United States has invested more than USD 110 million in ASD research networks and institutes over the past 20 years (excluding individual initiatives). There are still more clinical trials going on. The Autism Centers of Excellence (ACE) Centers and Networks programs continue these extensive programmatic NIH ASD research activities. The Innovative Medicines Initiative 2 (IMI2)-funded Multicenter Study for Developing New Medications (EU-AIMS; http://www.eu-aims.eu) is well underway, focusing on biomarker identification and individualized treatment regimens. Coupled with initiatives from privately financed organizations and industry, other cross-collaborative options, including Canada's POND network, have complemented and greatly aided the endeavor to collect complementary data. At the same time, smaller controlled studies of Oxytocin (Lemprière, 2022), Vasopressin (Parker et al., 2019; Bolognani et al., 2019), D-Cycloserine (Urbano et al., 2014; Minshawi et al., 2016; Wink et al., 2017), N-Acetylcysteine (Wink et al., 2016; Ghanizadeh and Moghimi-Sarani, 2013), CX516 (Berry-Kravis et al., 2006), Donepezil (Gabis et al., 2019; Handen et al., 2011; Buckley et al., 2011), and Fluoxetine (Lucchelli and Bertschy, 2018) have been completed, along with pharma-supported Phase II and III ASD. An increasing number of randomized controlled trials (RCTs) have focused on fundamental impairments, such as social withdrawal, in individuals with Arbaclofen treated for autism spectrum disorder (ASD) and fragile X syndrome (FXS). Additionally, many of these trials have targeted related symptoms, including irritability, hyperactivity, and anxiety, as their primary objectives. Although some businesses have deemed drug development for neurodevelopmental disorders (NDDs) to be too risky, a growing understanding of the neurobiology and genetics of ASD is causing this perspective to shift, thereby paving the way for the discovery of medicines for various behavioral endpoints in people with ASD (Ameis et al., 2018). Only three EMA and two US FDA indications have been approved thus far, and they are all for “irritability” or sleeplessness related to ASD.

Many molecules with different modes of action at different stages of clinical development are represented among the compounds now in development. A more comprehensive perspective that incorporates preclinical development of drugs is needed. Though Gene therapeutic models are picking up pace, they still have some bottlenecks that need to be addressed. Gene therapeutic agents need to be tested thoroughly at pre-clinical levels before being taken to clinical confirmatory trials. Optimum dose concentrations and copy number variants of genes must be investigated. Modes of gene regulation have to be examined, and inducers or gene modulators have to be utilized as and when required.

### Challenges in ASD clinical trials

Among the current difficulties in clinical trials for ASD are addressing the variability of ASD, failures to translate targeted medicines from preclinical to clinical, the absence of objective, validated biomarkers for target mechanism engagement, early change detection, therapy prediction, diagnosis, stratification, and pertinent brain circuit modulation, better clinical endpoints are required, setting molecular target priorities, developing stronger trial designs, managing the legal criteria for novel therapeutic uses, establishing priorities for comorbidity-focused therapies, including the viewpoints of participants and caregivers, and addressing potential ethical problems in research.

### Gene therapy

With just one or a few therapeutic interventions, gene therapies that permanently alter the genome could be a game-changing novel therapy for monogenic ASD. Transient gene therapies like RNA-editing, ncRNA, and ASOs leave the genome unblemished and will need repeated dosage, but it might have the benefit of being reversible and controlled (Weuring et al., 2021). These variations draw attention to the crucial questions surrounding gene therapies, specifically those related to dosage, delivery, and safety. However, better viral and non-viral vectors, such as delivery nanoparticles, more precise gene editors with minimal off-target effects, and regulated transgene expression, are made possible by the rapid speed of technological advancement (Weuring et al., 2021). The efficiency of a neuronal gene therapeutic strategy will rely on directing disease-modifying substances to the right positions, which incorporates both intracellular localization and transduction of the proper cells and circuits. Although liposomes or nanoparticle-mediated delivery are other options, a viral approach is most likely to be used to introduce a target gene or gene-editing tools into a neuron (Naldini, 2015). Leveraging the biology of the virus to express transgenes and altering or minimizing the residual viral genome in a way that inhibits pathogenic features like viral replication following host transduction are essential components of creating an efficient viral vector. Several genetically modified viruses under development can safely introduce heterologous genes or DNA sequences into neurons. Currently, lentiviruses and adeno-associated viruses (AAVs) are the two most common classes. Other viruses, such as HSV, rabies, and Semliki forest virus, can cause immunological reactions, transient transgene expression, or unacceptable toxicity in addition to transducing neurons (Manfredsson and Mandel, 2010).

AAV vectors' low toxicity, long-term gene expression, capacity to productively infect both dividing and non-dividing cells in a wide variety of host tissues and organs, and lack of pathogenesis are only a few of their many alluring qualities for safe and effective gene therapy (Berns, 1999; Kay et al., 2001). Several genetically modified viruses under development for the treatment of CNS disorders, AAVs, are currently the recommended gene delivery vector (Blessing and Déglon, 2016; Choudhury et al., 2017). They offer minimally harmful transgene expression over an extended period. Numerous AAV serotypes exist, and their distributions and tropisms can vary (Wu et al., 2006). Although it can occasionally integrate into the genome, AAV-delivered DNA is typically found in extrachromosomal episomes (High and Aubourg, 2011). Thus, insertional mutagenesis is possible, just like with lentiviruses. According to Zaiss and Muruve (2005), certain AAV serotypes exhibit a comparatively high level of natural immunity. AAV serotypes with low or no prevalence in the human population will be needed because neutralizing antibodies can stop brain transduction (Boutin et al., 2010). Investigations into using this vector should consider that the high levels of neutralizing antibodies (Nab) against wild-type AAV can decrease recombinant AAV (rAAV) mediated transduction in the brain (Peden et al., 2004). Anti-AAV antibody assay harmonization offers a chance to integrate immunogenicity data from multiple research projects, raise the degree of dependability for a crucial methodology, and generally enhance our capacity to forecast the effectiveness of viral vector-based gene therapies (GTx)—based therapy (Gorovits et al., 2021). The use of different analytical platforms and assay formats, the variety of reagents, and the semi-quantitative nature of total binding antibodies (Leiden Open Variation, n.d.) tests due to the absence of a real calibration standard are some of the difficulties that now impede standardization (Gorovits et al., 2021).

Several potential therapies are under investigation for ASD. A mouse model established the feasibility of using gene therapy to combat the loss of function of Scn2a channels and is under investigation as a therapeutic strategy for patients with ASD (Weuring et al., 2021). A hereditary form of ASD that has a SHANK3 mutation and deletion is the focus of JAG201, the gene therapy product which delivers a functional SHANK3 minigene, straight to CNS neurons via an adeno-associated virus serotype 9 (AAV9) vector is also under investigation (Nationa Library of Medicine, 2024).

As mentioned in the earlier section, overexpressing the human RORA protects neurons from death caused by oxidative stress (Boukhtouche et al., 2006c). Study shows that A2BP1, CYP19A1, HSD17B10, ITPR1, NLGN1, and NTRK2 are all regulated by RORA and when RORA levels are cut in half, the expression of all six genes are reduced in ASD (Sarachana and Hu, 2013). Additionally, RORA-deficient postmortem brain tissues from autistic individuals show lower expression levels of these six genes compared to age-matched, unaffected controls (Valerie, 2013). Furthermore, RORA alpha's transcriptional targets affect several directly regulated genes, including NLGN1 and NTRK2, as well as pathways that are disrupted in ASD (Devanna and Vernes, 2014). Recent research has linked the biology of autism to mutations in the X-linked neuroligin genes NLGN3 and NLGN4 (Ylisaukko-oja et al., 2005). There is evidence that *NTRK2* gene is a susceptibility factor for autism and a disruption of the BDNF/TrkB signaling pathway is associated with autism (Correia et al., 2010). Considering the above evidences showing the involvement of RORA in several pathways, AAV-hRORA-mediated gene therapy could hold promise to treat and minimize the effects of ASD as RORA transcriptionally regulates expression levels of several proteins, including aromatase (Sarachana et al., 2011), whose expression is reduced in ASD subjects. Pharmacologically stimulating RORA with synthetic agonists has demonstrated promising results in reducing repetitive behaviors in animal models. This suggests that restoring RORA function may be a part of treating ASD (Wang et al., 2016; Benger et al., 2018; Weuring et al., 2021). Although specific AAV-mediated hRORA gene therapy in humans is not yet well-documented in clinical studies, the efficacy of AAV-mediated gene therapies in other neurological illnesses and gene replacement models supports this approach as a reasonable future strategy (Ramachandran et al., 2025; Manini et al., 2022; Ye et al., 2024). Hence, further research is warranted on these grounds to optimize various studies at pre-clinical and clinical levels in ASD subjects, apart from understanding the mechanism of action in detail.

## New perspectives in NHR therapy: challenges and limitations

The importance of cellular context in NHR activity is shown by tissue-specific expression patterns and ligand availability (Evans and Mangelsdorf, 2014). Selective modulation techniques, like selective receptor modulators, may improve therapeutic specificity. Since the current medications often lack specificity and have serious side effects, like severe heart failure, researchers are currently developing new compounds with stronger binding affinities and improved specificity as novel NR-targeted medications (Huss and Kelly, 2004; Vega and Kelly, 2017). The NHRs, due to the effects that depend on tissue and context, as well as their varied functions, make it hard to carry out targeted treatments without causing unwanted side effects. Hepatotoxicity, cardiotoxicity, hormone disruption, cancer risk, metabolic disorders, and neurotoxicity are some of the dangers linked to unintended or prolonged regulation of NHRs (Rao et al., 2024). These harmful effects are particularly related to receptors such as glucocorticoid receptors, androgen receptors, estrogen receptors, and peroxisome proliferator-activated receptors (PPARs; Rao et al., 2024). To fully tap into the potential of NHRs, it is essential to understand their functions in different tissues, their connections with co-regulators, and their structural details. The current challenges in NHR research and drug development involve balancing their strong regulatory ability with concerns about specificity and safety (Schulman, 2010). NHR activation depends on specific ligands, which provide flexibility but also present risks of unwanted effects and multiple outcomes (Kanner, 1968). The lack of known endogenous ligands for several NHRs makes them “orphan receptors” (Rao et al., 2024). This complicates our understanding of their physiological roles and possible treatments. The natural disorder in certain NHR domains hampers full structural characterization and limits detailed understanding of their mechanisms. Drug targeting is also harder because the variable ligand affinity and competition among NHRs for co-regulators unpredictably affect receptor function. One of the most pressing scientific challenges in biomedicine is understanding how NHR genes are selected and creating specific inhibitors that target nuclear receptors, this warrants more structured studies.

### Conclusions

ASD is a serious condition that requires innovative treatment because of its complex physiology and lack of definitive cure. Understanding of ASD neurobiology, genetics, early identification, and early intervention have advanced significantly over the past 70 years since Leo Kanner initially identified autism (Kanner, 1943). With just one or a few treatments, gene therapies can potentially reduce the symptoms of the disorder. Antisense oligonucleotides, non-coding RNA, and RNA editing are examples of temporary gene treatments that leave the genome intact and necessitate repeated dosages, although they may have the benefit of being reversible and controllable. These variations draw attention to the crucial questions surrounding gene treatments, specifically the dose, delivery, and safety concerns, which should be thoroughly investigated for individual gene therapeutic drugs. There is an immense need to develop better viral and non-viral vectors for safe and effective delivery of gene therapy products. These include controlled transgene expression, more precise gene editors with minimal off-target effects, and nanoparticles for delivery. The speed at which accurate gene editing of a single-nucleotide gene mutation is getting close to therapeutic use is astounding. *In vitro* and *in vivo* testing of both temporary and permanent gene treatments for ASD has already been completed, paving the way for future drug development. Since structural variants necessitate a different strategy than missense mutations, the method of gene therapy is primarily determined by the precise genetic etiology. For the safe use of gene treatments in the future, accurate DNA diagnostics and accurate prediction of the functional effects of mutation will probably be equally crucial. In recent years, we have seen previously unheard-of breakthroughs in our understanding of neurology, genetics, early detection, and early intervention of ASD. Recent increases in ASD prevalence estimates underscore the critical need for continued work to translate new findings about ASD into effective interventions for all individuals with ASD. In this article, we highlight promising areas for new and existing research to speed scientific discovery and ultimately translate research findings into understood and empirically validated interventions for people with ASD. The relevance of RORA in ASD conditions can be corroborated through the fact that overexpression of human RORA confers neuroprotection by reducing the accumulation of ROS triggered by stress by increasing the production of the antioxidant proteins glutathione peroxidase 1 and peroxiredoxin 6, and the primary mediators of RORA's neuroprotective effects were glutathione peroxidase 1 and peroxiredoxin 6. Gene expression analysis of lymphoblastoid cell lines (LCLs) from people with and without ASD has confirmed that expression of RORA decreased in ASD, especially in the subtype linked to severe language impairment (Hu et al., 2006). Interestingly, exposure of cells to a methylation inhibitor can correct reduced expression, which is linked to increased methylation of the RORA promoter in LCL from people with ASD (Nguyen et al., 2010; Hu et al., 2006). Compared to sex- and age-matched controls, the post-mortem cerebellum and frontal cortex of people with ASD had lower levels of RORA protein. Further, male and female hormones regulate RORA in different ways, with estrogen raising RORA expression and dihydrotestosterone decreasing it (Sarachana et al., 2011). More importantly, RORA in turn controls the transcription of aromatase, an enzyme that changes testosterone into estrogen, offering a tenable explanation for the higher testosterone levels linked to a higher risk of ASD (Baron-Cohen et al., 2005), which brings in more strength to the theory of RORA being a formidable candidate in the treatment of ASD. Apart from the above facts, it was found that in the postmortem frontal brain of people with ASD, both RORA and aromatase proteins are significantly lower than in controls; a correlation coefficient of 0.91 indicates that there may be a causative link between RORA decrease and aromatase deficiency (Sarachana et al., 2011). And as per preliminary confocal immunofluorescence investigations, the frontal cortex of unaffected males has a 15–20% lower RORA protein level than that of age-matched females. This suggests sexual dimorphism in RORA expression. Based on the above facts, we tried to address autism spectrum disease and different therapeutic approaches, emphasizing the significance of AAV-hRORA-mediated gene therapy. Early diagnosis and personalized treatment for a variety of neurodegenerative diseases have improved due to the identification of novel biomarkers, such as cerebrospinal fluid (CSF), genetic markers, and developments in artificial intelligence (AI) and bioinformatics. Increased research focus in these areas may speed up the translation of knowledge about the etiological mechanisms of ASD to biological and psychological therapies to reduce the burden of ASD on affected individuals and their families.

### Future directions

In summary, recent increases in ASD prevalence highlight the urgent need to transform scientific progress into accessible, effective therapies. Future research must address barriers by exploring psychiatric comorbidities and overlaps with other neurodevelopmental disorders. Integrating behavioral, brain imaging, and genetic methods will help uncover mechanisms and individual differences within the ASD population. Emphasis should shift toward developmental trajectories and personalized interventions that respect neurodiversity and family needs. Additionally, identifying disease-modifying genes like NHRs could open new avenues for targeted treatments.
